# Contribution of cysteine residues in the extracellular domain of the F protein of human respiratory syncytial virus to its function

**DOI:** 10.1186/1743-422X-3-34

**Published:** 2006-05-24

**Authors:** Nicole D Day, Patrick J Branigan, Changbao Liu, Lester L Gutshall, Jianquan Luo, José A Melero, Robert T Sarisky, Alfred M Del Vecchio

**Affiliations:** 1 Department of Infectious Diseases Research, Centocor, Inc., 145 King of Prussia Road, Radnor, PA, 19087, USA; 2 Department of Structural Biology, Centocor, Inc., 145 King of Prussia Road, Radnor, PA, 19087, USA; 3 Centro Nacional de Microbiología, Instituto de Salud Carlos III, Majadahonda 28220, Madrid, Spain

## Abstract

The mature F protein of all known isolates of human respiratory syncytial virus (HRSV) contains fifteen absolutely conserved cysteine (C) residues that are highly conserved among the F proteins of other pneumoviruses as well as the paramyxoviruses. To explore the contribution of the cysteines in the extracellular domain to the fusion activity of HRSV F protein, each cysteine was changed to serine. Mutation of cysteines 37, 313, 322, 333, 343, 358, 367, 393, 416, and 439 abolished or greatly reduced cell surface expression suggesting these residues are critical for proper protein folding and transport to the cell surface. As expected, the fusion activity of these mutations was greatly reduced or abolished. Mutation of cysteine residues 212, 382, and 422 had little to no effect upon cell surface expression or fusion activity at 32°C, 37°C, or 39.5°C. Mutation of C37 and C69 in the F2 subunit either abolished or reduced cell surface expression by 75% respectively. None of the mutations displayed a temperature sensitive phenotype.

## Background

**Figure 1:**
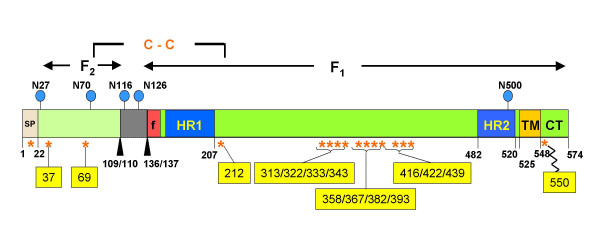
**Diagram of the HRSV F protein**. A linear representation of the HRSV F precursor protein (A2 strain) is shown.  Amino acid positions of individual domains are indicated with residues numbered in the context of the full-length coding region.  Disulfide linked F1 & F2 subunits are delineated with arrows.  The furin mediated cleavage sites are indicated by filed arrowheads.  The intervening cleavage fragment is indicated as a gray box.  Positions of the individual cysteine residues are depicted as asterisks.  Asparagine residues (N116 and N126) which are sites of N-linked glycosylation are represented with circles.  The site of palmitoylation at cysteine residue 550 is depicted as a jagged line.  SP = signal peptide; f = fusion peptide; HR1 = heptad repeat 1; HR2 = heptad repeat 2; TM = transmembrane region.  Figure adapted from [5].

Infection by HRSV is the single most common cause of hospitalization of infants and young children due to bronchiolitis and pneumonia and is a significant cause of morbidity and mortality the elderly and transplant recipients [1-4]. HRSV is member of the subfamily *Pneumovirinae *in the *Paramyxoviridae *family (reviewed in [5]. Three viral transmembrane proteins (F, G, and SH) are present on the surface of the virion particle [6]. The SH and G proteins are not required for virus replication in culture, although recombinant viruses lacking these genes are attenuated in animals [7-13]. The F protein is a type 1 membrane protein required for the fusion of the viral and host cell membranes as well as the formation of mature virion particles [10,14-16]. The HRSV F mRNA is translated into a 574 amino acid precursor protein designated F0, which contains a signal peptide sequence at the N-terminus that is removed by a signal peptidase in the endoplasmic reticulum (ER) [17-21]. F0 is contains 5 or 6 N-linked glycosylation sites depending upon virus strain [5,22,23]. F0 is cleaved at two sites [24] by furin in the *trans*-Golgi [18,19] removing a short, glycosylated intervening sequence and generating two subunits designated F1 (~50 kDa) that contains a single N-linked glycosylation site and F2 (~20 kDa) which contains two N-linked glycosylation sites [20]. The F1 and F2 chains are joined together by disulfide bond formation [25,26] although it has not been formally demonstrated which specific residues mediate this. The mature form of the F protein present on the surface of the virus and infected cells is believed to consist of a homotrimer consisting of three non-covalently associated units of F1-F2. This trimer has recently been shown to be quite thermostable [27]. Similar to other type I membrane viral fusion proteins (reviewed in [28], the F1 subunit contains a hydrophobic fusion peptide region followed by two heptad repeat regions (HR1 and HR2) that are separated by an intervening cysteine-rich region. A hydrophobic transmembrane domain is located near the C-terminus of the protein followed by a short (26 residues) cytoplasmic domain containing a single cysteine residue (Figure 1). Similar to other viral fusion proteins, F-mediated fusion with the host cell membrane is believed to be mediated by insertion of the fusion peptide into the host cytoplasmic membrane followed by subsequent conformational changes resulting in the interaction of the HR1 and HR2 regions, and the formation of a 6-helix bundle structure [29-31]. This process brings the viral membrane and host cell membrane in close proximity with each other allowing for lipid mixing and the fusion of the two membranes.

**Figure 2:**
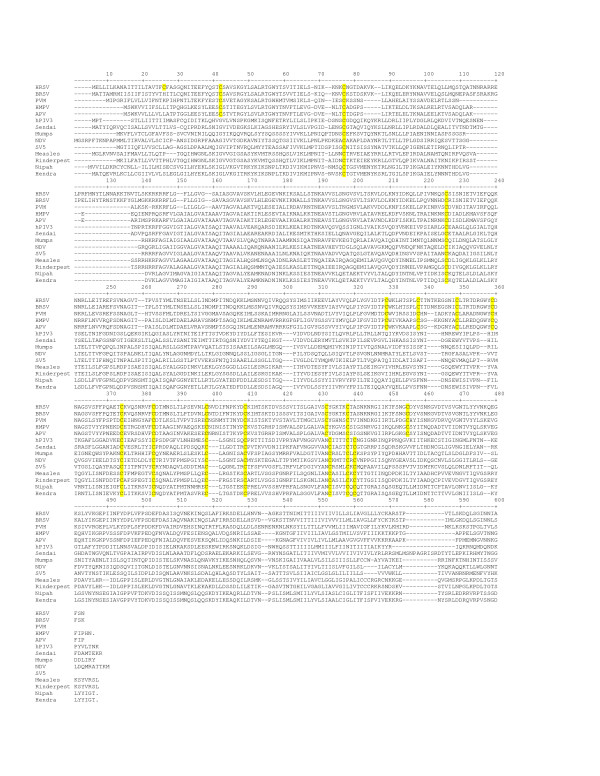
**Alignment of paramyxoviral F proteins**. Sequence alignment was performed as described in methods. Accession numbers for the sequences of the viral F proteins used for the alignment are as described in methods. Conserved cysteine residues are highlighted in yellow.

Although a structure of the crystal of the HRSV F protein 6-helix bundle has been determined [31] and electron microscopy images of HRSV F protein have been described [32], no detailed structural information for the entire protein exists. A partial x-ray structure of the somewhat distantly related Rubulavirus, Newcastle disease virus (NDV) F protein extracellular domain (ECD) [33,34] has been used to build a model of the HRSV F protein ECD [35,36]. More recently, the complete x-ray structure of the extracellular domain of the F protein of human parainfluenza virus 3 (hPIV3) has been solved [37]. The mature F protein of human respiratory syncytial virus (HRSV) contains fifteen cysteine residues that are absolutely conserved in all known isolates of both A & B subgroups of HRSV and BRSV and are highly conserved among the F proteins of the other Pneumoviruses such as pneumonia virus of mice (PVM), as well as in the Metapneumoviruses, human metapneumovirus (HMPV), and avian pneumovirus (APV) [38], and the F proteins of other paramyxoviruses including the well studied Newcastle disease virus (NDV) and Sendai virus [39,40] F proteins (Figure 2). No studies detailing the contribution of these cysteine residues to the structure or function of the HRSV F protein have been reported. The N-terminal signal peptide contains a single cysteine residue, however this region is removed by processing and is not present in the mature protein. A single cysteine residue is present in the cytoplasmic tail (position 550) has been shown to be the site of addition of a palmitoyl group in HRSV [41], although the cytoplasmic tail has been shown to not be required for cell fusion [42].

To determine the contribution of the individual cysteine residues in the extracellular domain (ECD) to its functions, a panel of mutations in which each cysteine residue in the ECD of the HRSV F protein (residues 37, 69, 212, 313, 322, 333, 343, 358, 367, 382, 393, 416, 422, 439) was individually changed to a serine, and the effect of these mutations upon the function of the HRSV F protein was determined.

## Results

**Figure 3:**
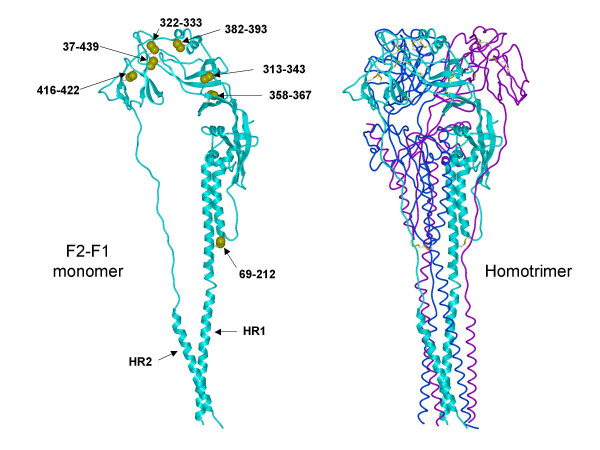
**Computer model of the HRSV F protein**. The molecular structure of HRSV F protein ECD was modeled using the human parainfluenza virus 3 virus F protein ECD structure as template as described in methods. Ribbon diagrams of the F1-F2 monomer (left) and F protein homotrimer (right) are shown. Heptad repeat 1 (HR1) and heptad repeat 2 (HR2) are indicated with arrows. Cysteine residues are depicted as yellow balls with specific residue disulfide pairs indicated on the monomer.

To better understand our results, the molecular structure of hRSV F protein was modeled using hPIV3 structure [pdb code 1ztm] [37] as a template. The sequence alignment was essentially the same as previously described [36] with a small adjustment of residues between 331 and 346 to allow all pairs of cysteine residues in the extracellular domain to be positioned close enough to form disulfide bonds. The trimer model of RSV F protein was constructed using Modeler software (Accelrys, CA) without further refinement. The resulting predicted disulfide bond pattern is 37–439, 69–212, 322–333, 313–343, 358–367, 382–393, and 416–422 (Figure 3).

**Figure 4:**
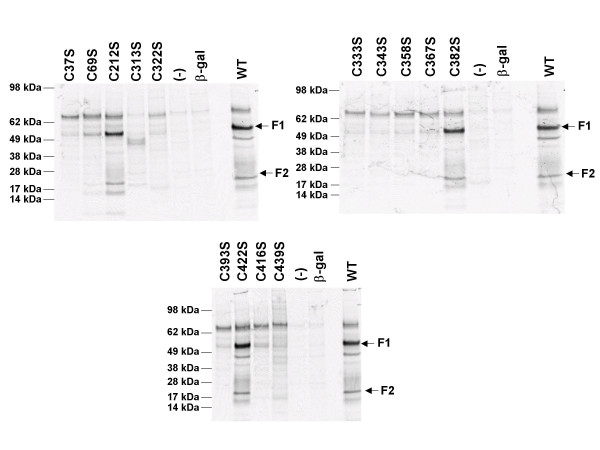
**Immunoprecipitation of HRSV F cysteine mutations**. 293T cells were mock transfected (-), transfected with a plasmid expressing beta-galactosidase (b-gal), or plasmids encoding the wild-type (WT) HRSV F protein or various cysteine mutants (listed above lanes), followed by metabolic labeling with [^35^S]-methionine/cysteine mixture, and immunoprecipitation as described in [44].  The positions of molecular weight size markers are indicated.  The positions of the F1 and F2 subunits are indicated with arrows.

To assess the effect of the cysteine mutations on protein expression, 293T cells were transfected with plasmids encoding either the wild-type F protein or those containing the individual cysteine mutations followed by metabolic labeling with [^35^S]-methionine-cysteine mixture. Cell lysates were prepared and immunoprecipitated with a cocktail of four anti-HRSV F mAbs (palivizumab, 47F, Mab19, and 101F) directed against the two major antigenic sites II and IV, V, VI [43] as previously described [44]. Levels of total immunoprecipitated F protein as well as the degree of cleavage of the F0 precursor into the F1 and F2 subunits were determined (Figure 4). A non-HRSV F related cellular band (present in lysates from cells transfected with empty vector (-) or beta-galactoside expression vector negative controls) that migrated slightly slower than the F0 precursor was also immunoprecipitated under these conditions. As shown in figure 4, mutation of extracellular cysteine residues 212, 382, 422 had little to no discernable effects on the levels of total immunoprecipitated protein or the degree of F0 cleavage relative to those observed for the wild-type HRSV F protein. Furthermore, the bands corresponding to the F1 and F2 subunits derived from these mutations migrated similarly to those from the wild-type HRSV F protein suggesting that these mutations had no gross effect on glycosylation. These findings are intriguing given that these three cysteine residues are absolutely conserved not only in the F proteins of other *Pneumovirinae*, but also in the F proteins of the *Paramyxovirinae *as well (Figure 2). In contrast, mutation of cysteine residues 37, 313, 333, 343, 358, 367, 393, 416, or 439 to serine all dramatically reduced or abolished the levels of total F protein immunoprecipitated as well as the degree of F0 precursor cleavage as determined by the levels of F1 and F2. These results suggest that either mutation of these cysteine residues to serine grossly affected the translation or folding of the F protein such that it was unstable or rapidly degraded, or that these mutations reduced the efficiency of binding of the four antibodies used in the immunoprecipitation. Based upon the model, residues 382 and 422 form disulfide bonds with residues 393 and 416 respectively. It is intriguing that mutation of one residue in the pair has no effect, while mutation of its bond partner residue has a dramatic effect. Together, these data would suggest that the formation of a disulfide bond between residues 382 and 393 or 416 and 422 is not required, but rather suggests the presence of a cysteine residue at positions 393 and 416 is critical. It is possible that loss of a disulfide partner in one case leads to aberrant disulfide bond formation by that free cysteine, while in the other case, the cysteine remains free and unbonded. Further work is needed to clarify the exact effect of such mutations. As these antibodies have been shown to recognize largely non-conformational epitopes [43,45], it would be unlikely to have a simultaneous loss of binding to both antigenic sites, thus we favor the interpretation that these cysteine mutations disrupted proper global protein folding and stability. Very low levels of F1 and F2 were observed with mutations C69S and C322S. Mutation C313S resulted in the appearance of a novel immunoprecipitating band migrating at approximately 45 kDa suggesting altered proteolytic cleavage or truncated translation. Further analysis is required to determine the exact nature of this band. Mutation of cysteine 69 to serine (C69S) reduced, but did not abolish expression or protein cleavage. These results suggest that mutation of cysteine residues 212, 382, and 422 did not disrupt folding sufficiently to affect processing of F0 to F1 and F2. Mutation of residues 69 and 322 dramatically reduced the levels of total protein immunoprecipitated as well the levels of F0 processed to F1 and F2. None of the mutations appeared to grossly affect glycosylation as the F0 and F1 and F2 subunits of all the cysteine mutations migrated similarly, although our gel system would not allow resolution of minor changes in glycosylation.

**Figure 5:**
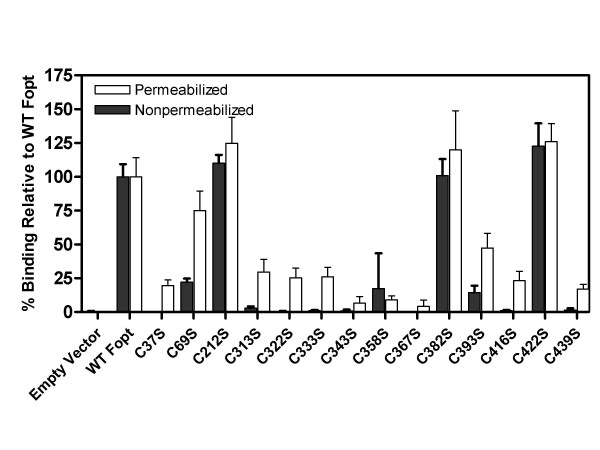
**Expression of cysteine mutations measured by ELISA**. 293T cells were transfected with plasmids encoding the wild-type HRSV F (WT), empty vector cassette (EV) or the various cysteine mutants (listed below lanes), followed by fixation and analysis using an ELISA as described in methods. Results are presented relative to values obtained with wild-type HRSV F which was set at 100%, and represent the average of three separate determinations. Results obtained using permeabilizing conditions are depicted with open bars. Results obtained using non-permeabilizing conditions are depicted with a solid bars.

To determine the role of the individual cysteine residues in cell surface expression, 293T cells transfected with plasmids expressing either wild-type HRSV F or the panel of cysteine mutations were analyzed by ELISA using palivizumab under either permeabilizing (to measure total protein) or non-permeabilizing (to measure cell-surface only) conditions. Values were calculated as percents relative to wild-type HRSV F after adjusting for background signal from the vector only control. As shown in Figure 5, cysteine mutations C212S, C382S, and C422S had similar levels of cell surface expression levels as wild-type HRSV F protein. Mutation of cysteine 69 to serine (C69S) reduced both total and cell surface expression by 25% and 72% respectively, but did not abolish expression or protein processing. Similar to the metabolic labeling results showing reduced total protein levels, mutations C37S, C313S, C322S, C333S, C343S, C358S, C367S, C393S, C416S, and C439S all had reduced levels of total protein (permeabilizing conditions) ranging from 49–92% reduction relative to wild-type F protein (Table 1). However, when the level of cell surface expression was examined by ELISA under non-permeabilizing conditions, all mutations had either low (8% for C393S, 3% for C313S) or no detectable levels of cell surface protein. This finding suggests that residues 37, 313, 322, 333, 343, 358, 367, 393, 416, and 439 are critical for cell surface expression most likely through their role in proper protein folding and disulfide bond formation. These results also suggest that the reduction in cell surface binding by the antibodies used in this study is not due to a diminished ability of these antibodies to recognize the cysteine mutations, as in several cases, F protein was clearly detected under permeabilizing conditions (Figure 5, C37S, C313S, C322S, C333S, C343S, C358S, C393S, C416S, C439S), but little to no F protein was detected under non-permeabilizing conditions. However, these results obtained using this assay can not rule out the possibility that in instances where cell surface F protein was not detected (under non-permeabilizing conditions), the protein encoded by these mutations was misfolded in such a way as to block the epitope recognized by the antibody.

**Table 1:** Summary of results for HRSV F cysteine mutants. Processing is defined as relative amounts of F0, F1, and F2, and is described as being equivalent to wild-type HRSV F protein (complete) or reduced. Cell surface and total expression were measured by ELISA under permeabilizing (total F protein) or non-permeabilizing (cell surface F protein) conditions using palivizumab as described in methods and reported as percent relative to wild-type HRSV F protein. Reactivity with neutralizing mAbs (palivizumab, Mab19, 47F, and 101F) as determined by flow cytometry is shown and reported as percent relative to wild-type HRSV F protein. Cell fusion activity is reported as luciferase activity measured at 32°C, 37°C, and 39.5°C as described in [44]. All values are expressed as % relative to wild-type at the respective temperatures.

	**Protein Processing**	**ELISA**		**Cell surface expression (Flow cytometry)**				**Cell fusion (% of WT)**		
		
		**Cell surface protein (Non-permeabilized)**	**Total protein (permeabilized)**	**Palivizumab**	**47F**	**101F**	**mAb19**	**32°C**	**37°C**	**39.5°C**
Wild-type	complete	100	100	100	100	100	100	100%	100%	100%
C37S	minimal	0.00	51	7	3	2	4	8%	8%	12%
C69S	reduced	25	72	22	19	21	20	10%	12%	12%
C212S	complete	117	103	37	43	46	39	52%	44%	34%
C313S	reduced	3	44	23	11	4	1	6%	5%	5%
C322S	minimal	0	35	5	1	3.5	0	7%	5%	6.5%
C333S	minimal	0	41	8	3	3	0.5	12%	8.5%	10%
C343S	minimal	0	22	4	0	1.5	0	14%	17%	19%
C358S	minimal	0	17	3	0	2	0	7%	4%	6%
C367S	minimal	0	8	8	3	9	1.5	10%	8%	9%
C382S	complete	103	90	86	102	96	88	105%	91%	100%
C393S	reduced	8	42	13	10	13	7	14%	10%	12%
C416S	minimal	0	43	4	3	5	0	5.5%	4%	4%
C422S	complete	141	132	90	93	81	81	140%	122%	146%
C439S	minimal	0.4	26	7	4	4	1	50%	29%	30%

To extend these results, the effect of the cysteine mutations upon the level of cell surface expression was examined by flow cytometry using four different antibodies, 47F [46], 101F (a monoclonal which recognizes the site IV, V, VI region), palivizumab [47] or mAb19 [48] directed against one of two major antigenic sites (II or IV, V, VI) in the F protein. Consistent with results obtained using ELISA under non-permeabilizing conditions, flow cytometry analysis demonstrated that mutation of cysteine residues 37, 313, 322, 333, 343, 358, 367, 393, 416, and 439 reduced binding of all four antibodies, while mutation of cysteine mutants C382S, and C422S retained similar levels of antibody binding as the wild-type F protein (Table 1). As the same set of cysteine mutations that reduced or abolished F0 protein cleavage and cell surface expression, also reduced or abolished cell surface binding of the four mAbs tested here, we conclude that cysteine residues 37, 313, 322, 333, 343, 358, 367, 393, 416, and 439 play a key role in the proper folding, processing, and cell surface transport of the HRSV F protein. Again, as the epitopes of these antibodies are directed against two different antigenic regions of F protein and have been shown to be largely non-conformational [43,45], we suggest that it is unlikely that the inability to detect these cysteine mutation F proteins on the cell surface is attributable to protein misfolding which would simultaneously block the epitopes recognized by these four different antibodies, but rather reflects a true defect in cell surface transport caused by these mutations. Interestingly, mutation of residue C212, which had wild-type levels of protein expression as determined by ELISA, appeared to have somewhat reduced levels of cell surface protein (37–47% of wild-type) as determined by flow cytometry. Although the exact reason for this is not clear, it could reflect a sensitivity of this particular mutant (folding, reactivity to fixation agent, etc.) to the differences in the experimental conditions used for ELISA and flow cytometry.

**Figure 6:**
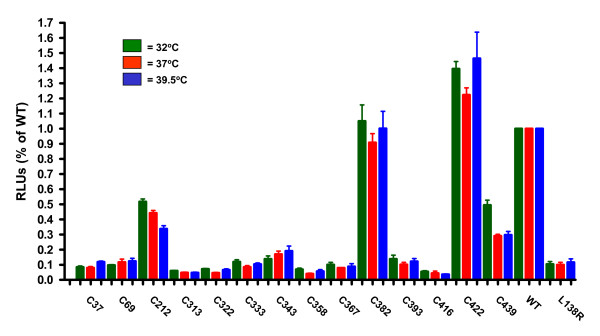
**Fusion activity of cysteine mutations**. 293T cells were transfected with plasmids encoding either the wild-type HRSV F protein or the panel of cysteine mutants and fusion activity was measured at 32°C, 37°C, or 39.5°C as described in [44]. Fusion activity is represented as relative light units (RLUs), and values represent the average of three separate determinations.

To assess the functionality of these cysteine mutations, a cell fusion assay was used as previously described [44]. As mutation of cysteine residues in other viral fusion proteins has been reported to cause a temperature-sensitive (*ts*) phenotype [49], we also examined the fusion activity of the panel of cysteine mutations at 32°C and 39.5°C as HRSV mutants sensitive for these two temperatures have been previously described [50,51]. The overall levels of wild-type HRSV F-mediated cell fusion are reduced by approximately 50% at either 32°C or 39.5°C relative to 37°C [42]. As shown in figure 6, mutation of cysteine residues 37, 69, 313, 322, 333, 343, 358, 367, 393, 416, and 439 reduced cell fusion activity to similar levels as a previously described point mutation in the fusion peptide region (pL138R) [44]. In contrast, mutations C382S and C422S had cell fusion activity equivalent to wild-type HRSV F protein. Mutation of cysteine residue 212 reduced fusion activity by 40–50%. This finding correlates with the reduced cell surface expression observed using flow cytometry. Although the absolute levels of HRSV F-mediated cell fusion were reduced at both 32°C and 39.5°C relative to 37°C for all proteins including wild-type (Figure 6), there were no differences observed in their relative fusion activities of the cysteine mutations at either 32°C and 39.5°C suggesting a lack of a gross *ts *phenotype for fusion for any of these mutations (Figure 4B).

## Discussion

Limited direct structure-function data exists for the HRSV F protein. This study utilizes a genetic approach to analyze the contribution of the individual cysteine residues in the extracellular domain in protein expression and cell fusion of the HRSV F protein and represents the first analysis of the contribution of the cysteine residues of the HRSV F protein ECD to its function. Generally, cysteine residues are critical for folding and provide structural stability to a protein via the formation of disulfide bonds. Mutation of cysteine residues 37, 313, 322, 333, 343, 358, 367, 393, 416, and 439 abolished or reduced cell surface expression to less than 7% of wild type HRSV F protein. This suggests that these residues play a key role in the proper folding and subsequent transport through the Golgi to the cell surface. Identification of the stages at which these specific cysteine mutations block the folding, maturation, and transport of the HRSV F protein is currently ongoing. Mutation of cysteine residues can often lead to a temperature sensitive (*ts*) phenotype such as that observed for the herpes simplex type 1 gD glycoprotein [49]. The lack of an observable *ts *phenotype in this study is supported by the high thermostability of the HRSV F protein among paramyxoviruses [27].

From direct mapping of disulfide bonds in Sendai virus [39], and based upon the positional conservation of the cysteine 69 residue in the HRSV F proteins with that of Sendai virus F protein and the F proteins from other of the *Paramyxoviridae*, it is likely that cysteine residues 69 and 212 participate in the disulfide linkage between the F1 and F2 subunits. The *Pneumovirinae *members have a positionally conserved second cysteine residue in the F2 subunit (corresponds to residue 37 in HRSV F protein) (Figure 2) not found in the other *Paramyxovirinae*. In the model of the HRSV F ECD, this cysteine residue is predicted to make a disulfide bond with cysteine residue 439, which is also only conserved in the F proteins of the *Pneumovirinae *members and not found in the F proteins of the other *Paramyxovirinae *members. This would suggest that two disulfide bonds are formed between the F1 and F2 subunits. We are currently performing direct biochemical mapping of the disulfide linkages to formally demonstrate this. This could explain, in part, the unique thermostability described for the HRSV F protein ECD [27].

HRSV is a significant human pathogen, and the F protein has been identified as the target of multiple neutralizing antibodies [47,52,53] as well as small molecule inhibitors [54-58]. As such, the HRSV F protein represents a critical viral target for the development of new and improved preventions and treatments for HRSV induced disease. A greater understanding of its structure-function relationships would greatly facilitate the development of these new agents. The results of this study provide further support that the highly conserved HRSV F protein cysteine residues play a critical role in the structure and function of this protein. As disulfide bonds have been shown to play roles beyond proper protein folding and stabilization of protein structure [59], it is tempting to speculate that, similar to HIV [60], the disulfide bonds of the Pneumovirus F proteins may have a direct role in fusion. Our modeling and analysis suggest the presence of two disulfide bonds which join the F1 and F2 subunits of the HRSV F protein. If formally demonstrated, this would highlight a distinct structural feature of the F proteins of the *Pneumovirinae *not described for the F proteins of the *Paramyxovirinae*.

## Methods

### Cells, plasmids and transfections

293T cells were grown at 37°C in a humidified atmosphere of 5% CO_2 _and maintained in Dulbecco's modified Eagle media (DMEM) with 4 mM L-glutamine adjusted to contain 1.5 g/L sodium bicarbonate, 4.5 g/L glucose and 10% FBS. Cells were tested and confirmed to be free of mycoplasma contamination. Plasmid pHRSVFoptA2, which expresses the HRSV F protein of the A2 strain whose sequence was codon optimized and derived from a known infectious HRSV cDNA [61], has been previously described [44] and served as the template for the generation of the panel of cysteine mutations by site directed mutagenesis using the QuikChange^® ^Site-Directed Mutagenesis kit (Stratagene^®^, La Jolla, CA). Cells were transiently transfected using FuGENE 6 reagent (Roche Applied Science, IN) as previously described [44].

### Metabolic labeling and immunoprecipitation

[^35^S]-methionine/cysteine radiolabeled cell lysates were prepared and immunoprecipitated with a cocktail of four anti-HRSV F mAbs (palivizumab, 47F, Mab19, and 101F) directed against the two major antigenic sites II and IV, V, VI [43] as previously described [44].

### ELISA

The binding of neutralizing monoclonal antibodies (mAbs) to HRSV F protein was assayed by ELISA using 293T cells transiently transfected with plasmids expressing either wild-type F protein, the panel of cysteine mutants, or a vector only control. 293T cells (2.0 × 10^4 ^cells/well) were plated the day before transfection in 96-well plates in DMEM, supplemented with 1.5 gms./liter sodium bicarbonate and 10% FBS. A total of 50 ngs of plasmid DNA was complexed with 0.15 μl of FuGENE 6 reagent and incubated 20 minutes room temperature in OptiMEM reduced-serum medium prior to addition to cells in serum containing medium. At 20–24 hours post-transfection, cells were assayed for binding of palivizumab under permeabilizing or non-permeabilizing conditions. Cells were fixed by the addition of 0.05% glutaraldehyde (Sigma) in 1X PBS for 15 minutes at room temperature. Cells were then either washed under conditions which permeabilizing (0.1% Triton-X100 in PBS) or non- permeabilizing (0.05% Tween-20 in PBS) conditions. These conditions were verified using an anti-RSV N protein mAb (clone # M291207, Fitzgerald Industries International, Concord, MA) and HRSV infected cells. HRSV N protein is only produced within the cytoplasm of HRSV infected cells. The anti-N mAb yields a strong positive signal on infected cells when the wash buffer containing 0.1% Triton-X100 is used, but not when wash buffer containing 0.05% Tween 20 is used (data not shown). Cells were blocked for one hour with SuperblockTM (Pierce Biotechnology, Inc., Rockford, IL) followed by incubation with either 1 μg/ml chimeric 101F IgG, 1 μg/ml palivizumab or a 1:600 dilution of mAb19 hybridoma supernatant for one hour at room temperature. Samples were then incubated with an anti-human IgG-HRP or an anti-mouse IgG-HRP as appropriate (Amersham Biosciences, Inc.) at 1:800 for one hour at room temperature followed by detection with TMB substrate (Sigma, Inc.). The reaction was stopped with the addition of 2N sulfuric acid, and the optical density at 450 nm was read. Values were calculated as percents relative to wild-type HRSV F after adjusting for background signal from the vector only control.

### Flow cytometry

To confirm cell surface expression, 293T cells were transfected with plasmids expressing either wild-type F protein, the panel of cysteine mutants or a vector only control in either 6-well or 96-well formats as described above. Cells were fixed with 2% paraformaldehyde in PBS for 15 minutes at 4°C. Cells were washed with PBS containing 2% FBS and then stained with either a chimerized human version of 101F (murine V region grafted onto human IgG1κ framework) or palivizumab (IgG1κ) at 1 μg/ml with an anti-human IgG-Alexa-Fluor-488 conjugated secondary (Molecular Probes, Eugene, OR) for analysis with the FACSCalibur (BD Bisociences) and determining the mean fluorescence intensity. Data analysis was performed with Cell Quest and FloJo Analysis Software. Values were calculated as percents relative to wild-type HRSV F after adjusting for background signal from the vector only control.

### Cell fusion assays

Cell fusion assays were conducted as previously described [44]. Briefly, one population of 293T cells was co-transfected with pHRSVFOptA2 and pBD-NFκB (effectors cells), and another population of 293T cells was transfected with the pFR-Luc luciferase reporter plasmid (reporter cells). At 24 hours post transfection, effector cells were mixed with an equal amount of reporter cells in a 96-well plate and incubated an additional 24 hours prior to measurement of luciferase activity using the Steady Glo Luciferase reporter system (Promega, Inc.).

### Computer modeling

The molecular structure of HRSV F protein ECD was modeled using the human parainfluenza virus 3 virus F protein ECD structure as template [pdb code 1ztm], essentially in the same way as previously described [36] with a small adjustment of the residues between 331 and 346, thus allowing all pairs of cysteine residues to be positioned close enough to form disulfide bonds. Sequence alignment was carried out in ICM (Molsoft, CA) and manually adjusted. The monomer molecular model was first generated in ICM and then the trimer was assembled.

### Sequence alignment

Sequence alignment was performed using the CLUSTAL W method in MegAlign program (version 5.05) from DNASTAR, Inc. (Madison, WI). Genbank accession numbers for the sequences of the viral F proteins used for the alignment are: HRSV [61], BRSV (NC_001989), PVM (AY729016), HMPV (NC_004148), APV (AY590688), hPIV3 (NC_001796), Sendai virus (NC_001552), Mumps virus (NC_002200), NDV (AF309418), Simian parainfluenza virus 5 (SV5) (NC_006430), Measles virus (P69353), Rinderpest virus (NC_006296), Nipah virus (NC_002728), Hendra virus (NC_001906).

## Competing interests

The authors PB, CL, ND, LG, JL, RS, and AD declare that are employees of Centocor, Inc. which provided supported for this work. JM is Director of the Centro Nacional de Microbiología Fundamental, Instituto de Salud Carlos III, and is a consultant for Centocor, Inc.

## Authors' contributions

PB, CL, and ND contributed equally to this work. PB and ND performed the ELISA assays, immunoprecipitations, and flow cytometry. CL generated reagents and performed the fusion assays. LG conducted site-directed mutagenesis of the HRSV F protein. JL generated the computer model of the HRSV F ECD. AD and RS participated in the design of the experiments, oversight of the conduct of the experiments, and AD, RS, and JM participated in the interpretation of the results.
